# Intercostal Cryoanalgesia Versus Thoracic Epidural Analgesia for Postoperative Pain Control After Thoracic Surgery: Study Protocol for a Randomized Clinical Trial

**DOI:** 10.2196/91837

**Published:** 2026-05-19

**Authors:** André Miotto, Juliana P Franceschini, Maria Luiza de Paula Pereira, Isabella Correia da Rocha, Maria Luiza Campos Ferreira, Suéllen Battaglini Rolando, Ernesto Evangelista Neto, João Aléssio Juliano Perfeito

**Affiliations:** 1 Departamento de Cirurgia, Disciplina de Cirurgia Torácica, Universidade Federal de São Paulo, Rua Botucatu, 740, Vila Clementino, São Paulo, São Paulo, 04023-062, Brazil, 55 11-914486014; 2 Fundação ProAR, Salvador, Bahia, Brazil; 3 Medical School, Universidade Nove de Julho, São Paulo, São Paulo, Brazil; 4 Escola Paulista de Medicina, Universidade Federal de São Paulo, São Paulo, São Paulo, Brazil

**Keywords:** intercostal cryoanalgesia, thoracic epidural analgesia, postoperative pain, thoracic surgery, pain management

## Abstract

**Background:**

Post-thoracic surgery pain remains a major clinical challenge, with substantial impact on pulmonary function, postoperative recovery, and patient quality of life. Thoracic epidural analgesia is widely regarded as the standard of care; however, it is associated with potential complications, including hypotension, urinary retention, and inadequate analgesia in a subset of patients. Intercostal cryoanalgesia, a peripheral nerve block technique that induces temporary axonal degeneration through controlled freezing, has emerged as a potential alternative for prolonged postoperative pain control.

**Objective:**

The primary objective of this study is to compare postoperative hospital length of stay between intercostal cryoanalgesia and thoracic epidural analgesia. Secondary objectives include the evaluation of postoperative pain intensity, opioid consumption, adverse effects, postoperative complications, quality of life, quality of recovery, and patient satisfaction.

**Methods:**

This is a single-center, prospective, randomized, parallel-group clinical trial comparing intercostal cryoanalgesia with thoracic epidural analgesia for postoperative pain control in patients undergoing thoracic surgery. Fifty adult patients (≥18 y) are randomized 1:1 to either epidural or cryoanalgesia groups. All perioperative and postoperative care is provided by the attending clinical teams according to routine institutional practice, with no influence from the research team beyond randomized allocation. The primary endpoint is postoperative hospital length of stay. Secondary outcomes include pain intensity (visual analog scale), opioid consumption, incidence of adverse effects and complications, quality of life (WHOQOL-BREF), and quality of recovery (QoR-15). Data are collected up to 1 year postoperatively.

**Results:**

Approval from the Human Research Ethics Committee was obtained in November 2024, and participant recruitment began in July 2025. Data collection commenced concurrently with participant recruitment and is expected to be completed by August 28, 2027. Data analysis will begin in September 2027, with results anticipated in the first quarter of 2028.

**Conclusions:**

This study protocol outlines a randomized clinical trial designed to assess clinical outcomes associated with intercostal cryoanalgesia compared with thoracic epidural analgesia following thoracic surgery. The findings are expected to contribute to the evidence base on postoperative pain management and inform the design of future comparative and implementation studies in this field.

## Introduction

Thoracic surgery is recognized as one of the most painful surgical procedures due to the extensive trauma to tissues, muscles, ribs, and intercostal nerves. Postoperative pain arises from a combination of nociceptive and neuropathic components and can significantly impair respiratory mechanics, mobility, and postoperative recovery [1,2]. Poorly controlled pain following thoracic surgery is associated with pulmonary complications such as atelectasis and pneumonia, prolonged hospitalization, and increased morbidity [3].

In addition, acute postoperative pain may progress to chronic pain syndromes, underscoring the importance of effective perioperative analgesic strategies [4,5]. Inadequate management of acute postoperative pain is a well-established risk factor for chronic post thoracotomy pain syndrome, a debilitating condition that may persist for months or even years after surgery [6-8]. Effective pain control, is therefore essential, for optimal postoperative recovery, with a direct impact on pulmonary function, mobility, and overall clinical outcomes [9].

Intravenous opioids remain a cornerstone of postoperative pain management; however, their use is associated with adverse effects including respiratory depression, nausea, vomiting, constipation, and dependence [6,10]. Thoracic epidural analgesia is considered the gold standard technique for open thoracotomy and other major thoracic procedures, providing superior analgesia and reduced pulmonary complications compared with systemic opioids [11,12]. Nevertheless, epidural analgesia carries inherent risks, such as hypotension, urinary retention, motor blockade, and potential neurological injury [13,14]. Furthermore, contraindications including coagulopathy and infection limit its applicability in selected patients [15].

Contemporary perioperative care increasingly favors multimodal analgesic strategies that combine systemic and regional techniques to optimize pain control while minimizing opioid exposure. Regional techniques such as intercostal and paravertebral nerve blocks have demonstrated efficacy in thoracic surgery [16,17,18]. However, continuous local anesthetic infusions delivered via indwelling catheters may be associated with complications including infection, catheter displacement, and pump malfunction [19].

Intercostal cryoanalgesia has re-emerged as a promising modality for postoperative pain control in thoracic surgery [20-23]. This technique induces a reversible interruption of sensory nerve conduction by freezing intercostal nerves to extremely low temperatures, typically between −70 °C and −90 °C, using compressed nitrous oxide. The resulting axonal and myelin sheath disruption inhibits nerve transmission while preserving the structural integrity of the endoneurium, perineurium, and epineurium, thereby allowing complete nerve regeneration over several weeks. This mechanism provides prolonged analgesia lasting approximately two months, covering the critical period of postoperative tissue healing, followed by full neural recovery [24-26].

Earlier studies comparing cryoanalgesia with intercostal nerve blocks or thoracic epidural analgesia suggested reductions in opioid consumption and hospital length of stay among patients receiving cryoanalgesia [27,28]. However, these studies were conducted using earlier-generation cryotechnology, limiting their applicability to current surgical practice. Advances in cryoanalgesia equipment, such as digital temperature control and ergonomic probes, have increased the precision and safety of the procedure. Therefore, the efficacy and safety of modern intercostal cryoanalgesia should be reassessed.

In this context, a prospective randomized clinical trial was designed to compare intercostal cryoanalgesia with thoracic epidural analgesia in adult patients undergoing thoracic surgery. The primary objective is to compare postoperative hospital length of stay between the two techniques. Secondary objectives include the evaluation of postoperative pain intensity, opioid consumption, adverse effects, postoperative complications, quality of life, quality of recovery, and patient satisfaction.

## Methods

### Study Design and Setting

This is a single-center, prospective, randomized, parallel-group clinical trial comparing intercostal cryoanalgesia with thoracic epidural analgesia for postoperative pain management in patients undergoing thoracic surgery. The study is conducted at the Thoracic Surgery Division of the Escola Paulista de Medicina, Federal University of São Paulo (UNIFESP), São Paulo, Brazil. Apart from randomization to the analgesic technique, no additional diagnostic or therapeutic interventions are mandated by the study protocol.

### Participants

Eligible participants are adults aged 18 years or older undergoing elective thoracic surgical procedures involving the pleural cavity, including: (1) anatomic lung resections (lobectomy, segmentectomy), (2) wedge resections, (3) pleural procedures, and (4) selected mediastinal surgeries. Surgical approaches include thoracotomy and video-assisted thoracoscopic surgery (VATS). Written informed consent is obtained from all participants prior to enrollment.

Exclusion criteria include procedures not involving access to the pleural cavity, sympathectomy, contraindications to thoracic epidural analgesia (such as coagulopathy or local infection), and cognitive impairment precluding informed consent or reliable pain assessment. Additional exclusion criteria include contraindications to cryoanalgesia, such as prior intercostal nerve injury in the target area, severe peripheral neuropathy, or conditions that may impair nerve regeneration.

### Randomization and Blinding

Participants are randomized in a 1:1 ratio to receive either intercostal cryoanalgesia or thoracic epidural analgesia. Randomization is performed using a computer-generated sequence with variable block sizes and stratification by surgical approach (VATS vs open surgery), prepared by an independent investigator. Allocation concealment is ensured using sequentially numbered, opaque, sealed envelopes opened immediately before surgery.

Stratification by surgical approach was implemented to account for potential differences in postoperative pain trajectories and recovery patterns between minimally invasive and open procedures. However, given the overall sample size, the study is not powered for formal interaction testing, and subgroup analyses will be considered exploratory.

Blinding of surgeons, anesthesiologists, and patients is not feasible due to the nature of the interventions. However, outcome assessors and data analysts remain blinded to group allocation, following a Prospective, Randomized, Open, Blinded Endpoint design to minimize assessment bias.

Given the open-label nature of the study and the inclusion of patient-reported outcomes (eg, pain, quality of life, and satisfaction), there is an inherent risk of reporting bias. To mitigate this limitation, outcome assessors and data analysts will remain blinded to treatment allocation, and standardized validated instruments will be used for all patient-reported measures.

### Interventions

In the control group, thoracic epidural analgesia is performed under aseptic conditions at an intervertebral level between T5 and T8, selected according to the planned surgical incision. The epidural space is identified using the loss-of-resistance technique, and catheter placement will be confirmed with 2% lidocaine. A continuous local anesthetic infusion will be maintained intraoperatively and postoperatively according to institutional protocols. Epidural failures, including inadequate sensory block, refractory hypotension, catheter malposition, or conversion to systemic analgesia, are documented and included in both intention-to-treat and per-protocol analyses.

The epidural analgesic regimen is not strictly standardized, as it follows institutional practice and clinician judgment. While this pragmatic approach enhances external validity, it may introduce variability in analgesic delivery, including the potential use of epidural opioids. This could influence total opioid consumption in the epidural group and will be acknowledged as a potential source of bias in the interpretation of results.

In the experimental group, intercostal cryoanalgesia is performed intraoperatively under direct visualization using the Cryo-S Painless system (Metrum Cryoflex, Poland). The cryoprobe, using pressurized nitrous oxide, achieves a probe-tip temperature of −89 °C and is applied directly to the intercostal nerves corresponding to the surgical incision site, including incision-level nerves and additional levels determined by the distribution of VATS ports, typically encompassing five intercostal levels. Each nerve will undergo a single freezing cycle of 2 minutes. Procedural parameters, including number of treated levels, freeze duration, probe temperature, and distance from the sympathetic chain, are prospectively documented to ensure procedural consistency.

### Postoperative Care and Follow-Up

All perioperative and postoperative clinical care is provided by the attending surgical and anesthesia teams in accordance with routine institutional practice for thoracic surgery. Decisions regarding analgesic medication selection, dosing, timing, and perioperative management, including preoperative preparation and postoperative care, are made exclusively by the treating physicians based on standard clinical judgment and patient-specific needs. The research team does not influence clinical decision-making beyond the randomized allocation to the analgesic technique under investigation. This approach is intended to reflect real-world clinical practice and to minimize performance and co-intervention biases.

Postoperative management follows standardized institutional protocols routinely used in thoracic surgery in both groups, including a multimodal analgesic regimen consisting of intravenous tramadol (100 mg every 8 h), oral codeine/paracetamol (30 mg every 6 h), or subcutaneous or intravenous morphine (2 mg every 4 h as needed for rescue analgesia). Non-opioid analgesics include dipyrone (1 g every 4‐6 h), paracetamol (500 mg every 6 h), ketoprofen (100 mg every 12 h), and gabapentin (300 mg every 12 h) unless contraindicated. Patient-controlled analgesia is initiated by the attending clinical team when predefined institutional thresholds for insufficient analgesia are met. Postoperative nausea, vomiting, pruritus, and sedation are recorded using standardized criteria. All patients receive respiratory physiotherapy and education regarding safe mobilization and analgesic use. Conversions and crossovers are prospectively documented and accounted for in intention-to-treat and per-protocol analyses.

Hospital discharge is determined by the attending clinical team according to standardized institutional criteria, including adequate pain control (visual analog scale, VAS ≤3 at rest), independent mobilization, stable respiratory status without supplemental oxygen, absence of fever for 24 hours, and chest drain removal according to institutional thresholds. Patients will be discharged with oral analgesics.

Postoperative follow-up assessments are conducted at one month, three months, and 12 months following the initial procedure. Outcomes include pain intensity (VAS at rest and during mobilization , such as coughing or ambulation), quality of life (World Health Organization Quality of Life – Brief Version, WHOQOL-BREF), quality of recovery (Quality of Recovery-15, QoR-15), physical activity, and postoperative complications. Screening for neuropathic pain (Pain Detection Questionnaire, painDETECT), and chest-wall sensory changes using von Frey monofilaments occur at three and 12 months.

### Outcomes

The primary outcome is postoperative hospital length of stay, defined as the number of days from surgery to hospital discharge. Secondary outcomes include pain trajectories, cumulative opioid consumption at 48 and 72 hours, time to chest drain removal, postoperative complications, opioid-related adverse effects, quality of recovery, health-related quality of life, chronic post thoracotomy pain at 3 and 12 months, ICU stay, and patient satisfaction. Adverse events are monitored and classified according to the Common Terminology Criteria for Adverse Events.

### Sample Size and Statistical Analysis

The sample size calculation was based on a superiority design, assuming a mean difference of 2 days in hospital length of stay between groups, a two-sided alpha of 0.05, and 90% power. Variability estimates were informed by prior literature [29]. A total of 46 participants (23 per group) is required. To account for potential losses and dropouts, a total of 50 participants will be enrolled.

Analyses will follow the intention-to-treat principle, with per-protocol analyses as sensitivity. The primary outcome will be analyzed using negative binomial regression, with adjustment for prespecified covariates, such as age, sex, American Society of Anesthesiologists physical status classification, smoking status, operative duration, and postoperative drain output. Secondary outcomes will be analyzed using appropriate parametric or nonparametric tests, mixed-effects models for repeated measures, and competing-risk analyses where applicable. Missing data will be addressed using multiple imputation. Statistical significance will be defined as a two-sided *P* value <.05. Adjusted analyses will include surgical approach (VATS vs open surgery) and procedure type (major vs minor) as prespecified covariates to account for potential confounding due to surgical heterogeneity.

Length of stay will be analyzed using negative binomial regression due to its expected skewed distribution and potential overdispersion. Although the sample size calculation was based on assumptions of mean differences derived from prior literature, this approach provides an approximation of the anticipated effect size, while negative binomial regression was selected to provide a more appropriate modeling strategy for count-type outcomes..

### Ethical Considerations

The study protocol has been reviewed and approved by the Research Ethics Committee of the Escola Paulista de Medicina, Federal University of São Paulo (UNIFESP), in São Paulo, Brazil. Prior to enrollment, all participants are provided with oral and written information regarding the study, and written informed consent is obtained from each participant.

The present trial has been prospectively registered in the Brazilian Registry of Clinical Trials (ReBEC, identifier RBR-78zfpxd) on March 1, 2025, prior to the enrollment of the first participant. The registered title differs slightly from the manuscript title due to administrative constraints, without changes to the study objectives, design, or outcomes. The study is conducted in accordance with the ethical principles of the Declaration of Helsinki, the Brazilian National Health Council Resolution 466/2012, and is reported following the SPIRIT (Standard Protocol Items: Recommendations for Interventional Trials) guidelines for clinical trial protocols. The CONSORT (Consolidated Standards Of Reporting Trials) principles will be followed for the future reporting of trial results.

Study data are collected and managed using REDCap (Research Electronic Data Capture), a secure, web-based platform hosted by UNIFESP. Access to the database is restricted to authorized study personnel, and data are stored in a deidentified format to ensure participant confidentiality. No identifiable personal data will be used in dissemination of the research.

## Results

Ethical approval for this study was obtained in November 2024. Participant recruitment began in July 2025 , and data collection commenced concurrently with recruitment. Follow-up assessments will continue until 12 months after the last enrolled participant, with final data collection expected to be completed by August 2027.

## Discussion

### Overview

Effective postoperative pain management is a cornerstone of enhanced recovery after thoracic surgery [30]. Although thoracic epidural analgesia provides effective analgesia and reduces pulmonary complications, its use is limited by contraindications, side effects, and technical complexity [11-15]. These limitations underscore the necessity to assess alternative analgesic strategies that maintain efficacy while enhancing safety and feasibility [31].

Intercostal cryoanalgesia represents a potential option within multimodal analgesic pathways [20]. Modern cryosurgical systems enable precise, reversible axonal disruption, providing prolonged regional analgesia without indwelling catheters or continuous infusions [6,32,33]. These technical advances address the shortcomings of older studies.

Recent evidence suggests that intercostal cryoanalgesia can effectively reduce postoperative pain intensity, opioid consumption, and length of hospital stay, while enhancing patient satisfaction and functional recovery [1,6,32,33]. Preliminary studies suggest that cryoanalgesia may yield favorable outcomes; however, high-quality randomized evidence comparing this approach with thoracic epidural analgesia remains limited.

By selecting hospital length of stay as the primary outcome, this study captures the integrated clinical impact of postoperative pain control on recovery. However, this outcome is multifactorial and may be influenced by surgical, institutional, and patient-related factors beyond analgesic strategy. Complementary secondary outcomes, including pain trajectories, opioid consumption, quality of recovery, and quality of life, will facilitate a more comprehensive and direct assessment of analgesic effectiveness. In addition, factors such as surgical complications, chest drain management, and discharge practices may influence length of stay. These factors will be accounted for through adjusted analyses including relevant perioperative variables, and through the interpretation of complementary secondary outcomes. Standardized documentation of cryoanalgesia parameters is intended to enhance reproducibility and facilitate future comparative and implementation studies.

Intercostal cryoanalgesia has theoretical and mechanistic features that support its evaluation as part of multimodal postoperative pain management strategies in thoracic surgery, including prolonged regional analgesia during the early postoperative period and the potential to reduce systemic opioid exposure. The systematic documentation of cryotherapy parameters, such as treated intercostal levels, achieved temperatures, and freeze–thaw duration, is intended to enhance reproducibility and enable detailed characterization of technique-related variability. This trial is designed to assess whether intercostal cryoanalgesia achieves postoperative outcomes comparable to those of thoracic epidural analgesia, thereby informing future clinical and implementation studies.

### Limitations

This study has some limitations inherent to its design. As a single-center trial, the findings may reflect local perioperative practices and patient characteristics, which could limit generalizability to other institutions. In addition, blinding of participants and care teams is not feasible due to the nature of the analgesic interventions, which may introduce performance or reporting bias, particularly for patient-reported outcomes.

The inclusion of a heterogeneous population encompassing different thoracic procedures and surgical approaches may introduce variability in outcomes such as pain and length of stay. Although randomization was stratified by surgical approach, residual heterogeneity may persist. To mitigate this limitation, relevant surgical variables will be incorporated into adjusted analyses, and exploratory subgroup analyses will be conducted, recognizing that the study is not powered to detect interaction effects.

Furthermore, the reliance on patient-reported outcomes in an open-label design may increase the risk of reporting bias, despite the use of validated instruments and blinded outcome assessment.

Nonetheless, the randomized design, standardized perioperative management, and prospective data collection are expected to mitigate these limitations and support the internal validity of the study.

### Dissemination

The results of this trial will be disseminated through publication in peer-reviewed scientific journals and presentations at national and international conferences. Summaries of findings will also be shared with participating patients upon study completion.

### Conclusion

This randomized clinical trial is designed to provide prospective, methodologically rigorous evidence comparing intercostal cryoanalgesia and thoracic epidural analgesia for postoperative pain management in thoracic surgery. By integrating clinical outcomes, patient-reported measures, and recovery-related endpoints within a standardized perioperative pathway, the study aims to clarify the role of cryoanalgesia as part of multimodal analgesic strategies. The findings are expected to inform clinical decision-making and support the evidence-based incorporation of cryoanalgesia into contemporary thoracic surgical practice, particularly in settings where epidural analgesia may be contraindicated or less desirable.

## Abbreviations

CONSORT: Consolidated Standards Of Reporting Trials
REDCap: Research Electronic Data Capture
SPIRIT: Standard Protocol Items: Recommendations for Interventional Trials
UNIFESP: Federal University of São Paulo
VAS: visual analog scale
VATS: video-assisted thoracoscopic surgery
